# The Association among Difficulties in Emotion Regulation, Hostility, and Empathy in a Sample of Young Italian Adults

**DOI:** 10.3389/fpsyg.2016.01068

**Published:** 2016-07-18

**Authors:** Anna Contardi, Claudio Imperatori, Ilaria Penzo, Claudia Del Gatto, Benedetto Farina

**Affiliations:** Department of Human Science, European University of Rome, RomeItaly

**Keywords:** emotion regulation, hostility, empathy, difficulties in emotion regulation, mediational model

## Abstract

The aim of the present study was to assess the role of empathy in mediating the association between difficulties in emotion regulation and hostility. Three hundred and sixty young Italian adults (220 women and 140 men) were enrolled in the study. Psychopathological assessments included the Difficulties in Emotion Regulation Scale (DERS), the Interpersonal Reactivity Index and the Buss–Durkee Hostility Inventory (BDHI). Perspective taking (PT) and Personal distress (PD) are significantly associated with both DERS total score and BDHI total score. A mediational model analyzing the direct and indirect effects of DERS on BDHI through the mediating role of PT and PD showed that the relation between DERS and BDHI was partially mediated by PT total score (*b* = 0.16; *se* = 0.01; *p* = 0.02). Taken together our findings support the possibility that PT skills could play a crucial role in inhibiting hostility behaviors.

## Introduction

Emotion regulation consists of *“the extrinsic and intrinsic processes responsible for monitoring, evaluating, and modifying emotional reactions, especially their intensive and temporal features, to accomplish one’s goals”* (Thompson, 1994). This involves: (1) emotional clarity, awareness, and acceptance; (2) the capacity to control impulsive behaviors when feeling negative emotions; (3) the ability to choose contextually suitable emotion regulation strategies in order to meet personal goals and situational demands (Gratz and Roemer, 2004).

In recent years, difficulties in emotion regulation have been increasingly associated with the development and maintenance of several mental-health problems and maladaptive behaviors (Amstadter, 2008; Gillanders et al., 2008; Aldao et al., 2010; Jimenez et al., 2010; Marroquín, 2011; Aldao and Mennin, 2012; Berking et al., 2012; Svaldi et al., 2012). Furthermore, it has been observed (Berking and Whitley, 2014) that difficulties in emotion regulation are detected in almost all mental disorders included in the 5th edition of Diagnostic and Statistical Manual of Mental Disorders (American Psychiatric Association, 2013). For example, difficulties in emotion regulation seem to be related with both internalizing (e.g., major depression and anxiety disorders) (Mennin et al., 2007; Nolen-Hoeksema et al., 2008; Contardi et al., 2013) and externalizing behavior problems (e.g., attention-deficit/hyperactivity disorder) in adolescents and young adults (for a review see Steinberg and Drabick, 2015).

Among the behavioral correlates of the difficulties in emotion regulation, expressing hostility has been particularly investigated in both clinical and non-clinical settings (Bowie, 2010; McLaughlin et al., 2011; Carrère and Bowie, 2012; Mitrofan and Ciuluvică, 2012; Roberton et al., 2012). Hostility is conceptualized as a multidimensional construct including cognitive (i.e., negative thoughts, cynicism, or resentment), affective (i.e., negative emotions, including distaste, and anger), and behavioral components (i.e., verbal and physical aggression) (Garcia-Leon et al., 2002). A recent review of longitudinal studies investigating the association between emotion regulation and aggressiveness in adolescents suggested that deficits in emotion regulation are an important risk factor for aggressive behaviors (Roll et al., 2012). Similarly, a lower emotion regulation predicted subsequent relationship aggressiveness (Bowie, 2010). Moreover, Mitrofan and Ciuluvică (2012) reported that several components of emotion regulation (i.e., acceptance of emotions, ability to control impulses) should be enhanced in order to reduce the expression of anger and hostility as well as to increase life satisfaction in adolescents.

Both difficulties in emotion regulation and hostility seem to be closely related with trait (or dispositional) empathy (Jolliffe and Farrington, 2004; Decety, 2010). As defined by Davis (1994), dispositional empathy is a multidimensional construct with both emotional (i.e., the tendency to worry or feel solidarity with others) and cognitive (i.e., the tendency to identify with others and take into consideration their point of view) components. It has been proposed that emotion regulation may be one of the core components (together with affective arousal and emotion understanding) of human empathy (Decety, 2010). Furthermore, experimental studies reported that both cognitive and emotional components of empathy are related with emotion regulation (Eisenberg et al., 1994; Okun et al., 2000).

Similarly, the association between empathy and hostility has been widely detected. Low levels of empathy may be positively associated with more aggressiveness and disruptive behavior disorders (Jolliffe and Farrington, 2004; de Wied et al., 2010). Furthermore, higher levels of empathy increase prosocial behaviors (Davis et al., 1994; McMahon et al., 2006; Gini et al., 2008; Masten et al., 2011) and moderate the expression of aggressive behaviors and different types of violence, such as delinquent bullying behavior, alcohol-related and sexual aggressions (Wheeler et al., 2002; Giancola, 2003; Lovett and Sheffield, 2007; Jolliffe and Farrington, 2011).

Despite the strong association between difficulties in emotion regulation and hostility, no study so far has investigated the association between these two constructs while considering the possible role of empathy as a mediating factor. Therefore, the aim of the present study was to investigate in a sample of young Italian adults (i) the association between self-reported difficulties in emotion regulation and hostility, and (ii) whether this association was mediated by self-reported deficits in empathy. We decided to focus on this developmental stage because it is known that the onset of several mental disorders, characterized by severe emotion dysregulation, such as addictive disorders and impulse control disorders, occurs in young adulthood (Christie et al., 1988; Jones, 2013). We hypothesized that more severe difficulties in emotion regulation were associated with increased hostility, and that this association was partially mediated by empathy.

## Materials and Methods

### Participants and Procedure

Participants were 360 young Italian adults (220 women and 140 men). Inclusion criteria were: (i) age range between 18 and 34 years, (ii) good ability to understand written Italian. Exclusion criteria were the presence of factors that impeded complete assessment, such as the refusal of informed consent. The sample was recruited through accidental sampling. University psychology students (*N* = 267) were enrolled at the European University of Rome and completed the assessment during normal academic activities at their teaching sites. The non-university sample (*N* = 93) was recruited through advertisements for established community groups (e.g., hospitals, shopping malls, church groups operating in Rome).

Participation rate was 98%. There were no sociodemographic differences between responders and non-responders as well as between university students and the non-university sample. All subjects participated voluntarily and anonymously in the study after providing written informed consent; they did not receive payment or any other compensation (i.e., academic credit).

Mean age of the respondents was 23.17 years (*SD* = 3.72). Of the respondents, 23.6% had a college degree, while the remaining were middle and high school graduates (no one attended school for less than 8 years). Around 94% were single, 3.6% were married, and 2.5% were either widowed or divorced. Other characteristics of the sample are reported in **Table 1**.

**Table 1:** Descriptive statistics of all participants (*N* = 360).

Variables	Count/M	%/(*SD*)
Females	220	61.1
Age -*M (SD)*	23.17	(3.72)
Job status		
Employed	93	25.8
Students	267	74.2
School attainment > 13 years	85	23.6
Marital status		
Unmarried	338	93.9
Married	13	3.6
DERS total scores -*M (SD)*	86.91	(20.70)
Non-acceptance -*M (SD)*	13.34	(4.80)
Goals -*M (SD)*	14.11	(4.41)
Impulse -*M (SD)*	14.04	(5.12)
Awareness -*M (SD)*	15.20	(4.37)
Strategies -*M (SD)*	18.26	(6.33)
Clarity -*M (SD)*	11.95	(4.10)
IRI		
FS -*M (SD)*	2.18	0.67
EC -*M (SD)*	2.70	0.54
PT -*M (SD)*	2.31	0.64
PD -*M (SD)*	1.62	0.64
BDHI -*M (SD)*	35.16	(10.10)
Covert -*M (SD)*	10.89	(4.50)
Overt -*M (SD)*	24.26	(7.50)

*DERS, Difficulties in Emotion Regulation Scale; IRI, Interpersonal reactivity index; FS, Fantasy; EC, Empathic concern; PT, Perspective taking; PD, Personal distress; BDHI, Buss–Durkee hostility inventory.*

After receiving information about the aims of the study, subjects provided written consent to participate in the study, which was performed according to the Helsinki declaration standards and was approved by the European University’s ethics review board.

### Measures

The Italian versions of the Difficulties in Emotion Regulation Scale (DERS) (Gratz and Roemer, 2004; Sighinolfi et al., 2010), the Interpersonal Reactivity Index (IRI) (Davis, 1980, 1983; Albiero et al., 2006), and the Buss–Durkee Hostility Inventory (BDHI) (Buss and Durkee, 1957; Castrogiovanni et al., 1993) were administered in the present study.

The DERS is a 36-item multidimensional self-report measure assessing individual’s characteristic patterns of emotion regulation. Items are rated on a 5-point Likert-type scale (from 1 = *almost never* to 5 = *almost always*) indicating the degree to which each statement describes the respondent’s behavior. Scores range from 36 to 180; greater scores on the DERS reflect greater difficulties with emotion regulation. This test consists of the following six subscales, theoretically formulated and confirmed through factor analysis: (1) *Non-acceptance*, referred to non-acceptance of emotion responses (e.g., “When I’m upset, I feel guilty for feeling that way”); (2) *Goals*, related to the difficulty in engaging in a goal-directed behavior while experiencing negative emotions (e.g., “When I’m upset, I have difficulty concentrating”); (3) *Impulse*, referring to the impulse control difficulty when experiencing negative emotions (e.g., “When I’m upset, I have difficulty controlling my behaviors”); (4) *Awareness*, related to emotional awareness (e.g., “I am attentive to my feelings”); (5) *Strategies*, concerning the limited access to emotion regulation strategies that are perceived as effective (e.g., “When I’m upset, I start to feel very bad about myself”); and (6) *Clarity*, related to the lack of emotional clarity (e.g., “I’m confused about how I feel”). The DERS showed a good level of internal consistency for both total score (Cronbach’s α = 0.93) and the six subscales (Cronbach’s α > 0.80) (Gratz and Roemer, 2004). The instrument also revealed an adequate concurrent validity with measures of emotion dysregulation and emotional avoidance, as well as a good predictive validity with behaviors associated with emotion dysregulation, such as self-harm and marital violence (Gratz and Roemer, 2004). In the present sample Cronbach’s α for the DERS total score was 0.91.

The IRI is a 28-item self-report measure of dispositional empathy. Each item is rated on a 5-point Likert-type scale, ranging from 1 (*Does not describe me well*) to 5 (*Describes me very well*). Scores range from 28 to 140; greater scores on the IRI reflect greater dispositional empathy. The IRI measures four dimensions of empathy: (1) *Perspective taking* (PT), measuring the reported tendency to spontaneously adopt the psychological point of view of others in everyday life (e.g., “I sometimes try to understand my friends better by imagining how things look from their perspective”); (2) *Empathic concern* (EC), measuring the tendency to experience feelings of sympathy and compassion for unfortunate others (e.g., “I often have tender, concerned feelings for people less fortunate than me”); (3) *Personal distress* (PD), assessing the tendency to experience severe discomfort in response to extreme distress in others during a tense emotional situation (e.g., “In emergency situations, I feel apprehensive and ill-at-ease”); (4) *Fantasy* (FS) measuring the tendency to imaginatively transpose oneself into fictional situations (e.g., “I daydream and fantasize, with some regularity, about things that might happen to me”). Although several self-report measures of empathy have been developed (for a review see Pedersen, 2009), currently the IRI is the most widely and frequently used scale to measure individual differences in empathic tendencies (Spreng et al., 2009). We decided to use IRI because it is based on a multidimensional conceptualization of empathy and it is considered the most comprehensive measure of self-reported empathic dispositions (De Corte et al., 2007; Ingoglia et al., 2016). Finally, under the psychometric point of view, the IRI is characterized by several good psychometric properties, such as good internal consistency (Davis, 1994) as well as high replicability of the four-factor model in many countries (Ingoglia et al., 2016), including Italy (Albiero et al., 2006). In the present sample all IRI dimensions had Cronbach’s α of 0.78 or higher.

The BDHI consists of 75 dichotomous items (i.e., true–false answers). It was specifically developed to tap seven different subtypes of hostility (66 items) and guilt (9 items). Scores range from 28 to 140; greater scores on the BDHI reflect greater hostility. The dimensions of the BDHI, based on a theoretical classification of subtypes of hostility, are: (i) *Assault* (e.g., “If somebody hits me first, I let him have it”); (ii) *Indirect Hostility* (e.g., “I sometimes spread gossip about people I don’t like”); (iii) *Irritability* (e.g., “I often feel like a powder keg ready to explode”); (iv) *Negativism* (e.g., “When someone is bossy, I do the opposite of what he asks”), (v) *Resentment* (e.g., “Almost every week I see someone I dislike”); (vi) *Suspicion* (e.g., “I know that people tend to talk about me behind my back”); (vii) *Verbal Hostility* (e.g., “If someone annoys me, I am apt to tell him what I think of him”). Factor analysis of the BDHI has yielded two factors, one related to overt expression of hostility, generally consisting of *Assault*, *Indirect Hostility*, *Irritability* and *Verbal Hostility* and the other linked to covert expression of hostility, consisting of *Resentment* and *Suspicion* subscales (Buss and Durkee, 1957; Sarason, 1961; Bendig, 1962; Musante et al., 1989; Bushman et al., 1991). Two-week test-retest reliability coefficients have been reported to range from 0.64 to 0.78 for the subscales, and to be 0.82 for the total score (Biaggio et al., 1981). The BDHI revealed a good convergent validity with other self-report measures of anger, hostility, and aggression (Matthews et al., 1985). In the present sample Cronbach’s α for the BDHI total score was 0.85.

### Statistical Analysis

Relationships between variables were computed through Pearson’s indices of associations (*r*).

To determine whether the relationship between difficulties in emotion regulation and hostility severity was partially mediated by empathy, we used the Preacher and Hayes’ (2008) strategy, which assesses *“how, or by what means, an independent variable (X) affects a dependent variable (Y) through one or more potential intervening variables, or mediators (M)”* (Preacher and Hayes, 2008, p. 879).

This strategy tests mediation with a product-of-coefficients approach via a series of regressions analysis (Pompili et al., 2015). In the present analyses, we used standardized variables to generate standardized coefficients and the corresponding *p* values. As suggested by Preacher and Hayes (2008), for indirect effects, we also calculated bias-corrected and accelerated 95% CI produced using a bootstrapping method.

In the present study, we tested a model in which hostility severity (BDHI total score) was the dependent variable and difficulties in emotion regulation (DERS total score) were the independent variable. IRI dimensions, significantly associated with both the DERS and the BDHI at the bivariate analyses, were examined as a potential mediator. Additionally, we included age and gender in the model, which are known to be related with both emotion regulation (Blanchard-Fields et al., 2004; Nolen-Hoeksema, 2012) and hostility (Barefoot et al., 1993; Davidson and Hall, 1995). In order to test the adequacy of the model, we have also performed a reverse mediational model, in which hostility is the independent variable and difficulties in emotion regulation is the dependent variable.

It should be noticed that in the mediational models, the relations between variables are supposed to be causal, and mediational processes usually develop over time (Pompili et al., 2015). For this reason, several researchers questioned the use of cross-sectional data in mediation models. However, it is also argued that the use of prospective studies does not always prove causality (Hayes, 2013). Furthermore, according to Salthouse (2011), mediation strategies can also be viewed as a type of variance partitioning, similar to other methods (e.g., partial correlation), and they can also be useful when investigating whether the relation between two variables is reduced when a mediating variable is considered.

All analyses were performed with the statistical package for the social sciences (SPSS) version 19.0 (IBM, Armonk, NY, USA) and the macro for SPSS Indirect (Preacher and Hayes, 2008).

## Results

In the present sample the mean score of DERS, BDHI, and IRI subscales were comparable to those reported in previous studies which investigated these variables in non-clinical subjects having similar socio-demographic characteristics to our sample (Fossati et al., 2004; Albiero et al., 2006; Giromini et al., 2012).

### Associations among Difficulties in Emotion Regulation, Hostility, and Empathy

Correlations between variables are reported in **Table 2**. The DERS total score was positively and strongly associated with the BDHI total score (*r* = 0.51; *p* < 0.001). The DERS total score was also positively associated with FS (*r* = 0.12; *p* = 0.02) and PD (*r* = 0.38; *p* < 0.001) total score, and negatively associated with PT total score (*r* = -0.13; *p* = 0.01). PD (*r* = 0.17; *p* = 0.002) and PT (*r* = -0.31; *p* < 0.001) were also associated with the BDHI total score.

**Table 2:** Association between the DERS, the BDHI, and IRI dimensions (*N* = 360).

	DERS	FS	EC	PT	PD	BDHI
DERS	-					
FS	0.12*	-				
EC	-0.05	0.33***	-			
PT	-0.13*	0.30***	0.44***	-		
PD	0.38**	0.25***	0.15**	0.20	-	
BDHI	0.51***	0.07	-0.10	-0.31***	0.17**	-

*p < 0.05; **p < 0.01; ***p < 0.001. DERS, Difficulties in Emotion Regulation Scale; FS, Fantasy; EC, Empathic concern; PT, Perspective taking; PD, Personal distress; BDHI, Buss–Durkee hostility inventory.

**FIGURE 1:**
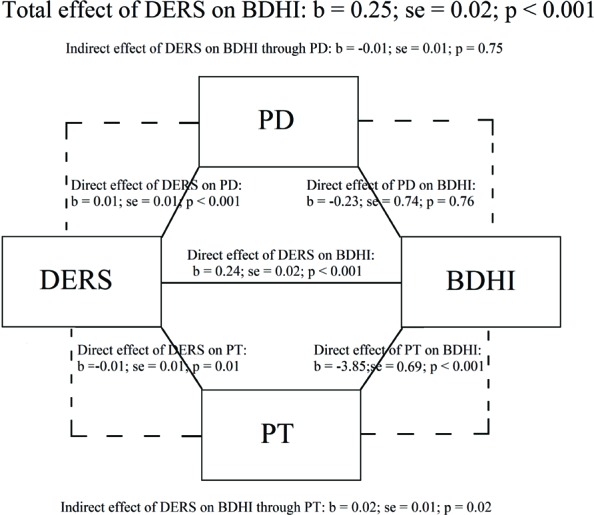
**Test of effect of difficulties in emotion regulation on hostility through perspective taking (PT) and personal distress (PD)**.

The mediational model explained 30% of data variability (*F*_5,354_ = 33.88; *p* < 0.001). Preacher and Hayes’ (2008) strategy indicated that the total effect of the DERS on the BDHI was significant (*b* = 0.25; *se* = 0.02; *p* < 0.001), with more severe difficulties in emotion regulation being associated with more severe hostility (**Figure 1**). Moreover, the relationship between difficulties in emotion regulation and hostility was partially mediated only by PT, with higher scores on the DERS being associated with lower PT scores, which were associated with higher BDHI scores [*b* = 0.02; *se* = 0.01; *p* = 0.02; (95% CI: 0.01/0.04)]. No significant effect was observed for PD [*b* = -0.01; *se* = 0.01; *p* = 0.75; (95% CI: -0.02/0.02)]. Age and gender had no effects on BDHI total score (Age: *b* = 0.19; *se* = 0.12; *p* = 0.88; Gender: *b* = -0.40; *se* = 0.99; *p* = 0.69). A second mediational model with only PT as mediator was also significant explaining 32% of data variability (*F*_4,355_ = 42.41; *p* < 0.001).

Finally, the reverse mediational model, with BDHI total score as the independent variable and DERS total score as the dependent variable, explained 27% of data variability (*F*_4,355_ = 34.58; *p* < 0.001). Although Preacher and Hayes’ (2008) strategy indicated that the total effect of the BDHI on the DERS was significant (*b* = 1.03; *se* = 0.09; *p* < 0.001), no significant mediational effect was observed for PT [*b* = -0.02; *se* = 0.03; (95% CI: -0.08/0.04)].

## Discussion

The main aim of the present study was to assess the association between emotion regulation and hostility, exploring the role of empathy as a ‘mediator.’ Our results showed that: (i) difficulties in emotion regulation are positively associated with hostility, (ii) personal distress dimension is positively related with both difficulties in emotion regulation and hostility, (iii) perspective taking dimension is negatively associated with both difficulties in emotion regulation and hostility, (iv) fantasy dimension is positively related with difficulties in emotion regulation but not with hostility, and (v) more severe difficulties in emotion regulation are associated with increased severity of hostility, and this association was partially counterbalanced only by the mediational effect of perspective taking.

The association between difficulties in emotion regulation and hostility has been consistently detected in both clinical and non-clinical settings (Bowie, 2010; McLaughlin et al., 2011; Carrère and Bowie, 2012; Mitrofan and Ciuluvică, 2012; Roberton et al., 2012). For example, Roll et al. (2012), reviewing longitudinal studies, investigating the relationship between emotion regulation and aggressive behavior in childhood, concluded that emotion dysregulation is an important risk factor for aggressive behaviors (Roll et al., 2012). In line with previous data, our results also showed that empathy dimensions (i.e., personal distress and perspective taking) were related with both difficulties in emotion regulation (Eisenberg et al., 1994; Okun et al., 2000) and hostility (Gini et al., 2007; Fernández et al., 2011; Day et al., 2012). Previous data showed that low levels of empathy are positively associated with more aggressive behaviors and disruptive behavior disorders (Jolliffe and Farrington, 2004; de Wied et al., 2010). Conversely, higher levels of empathy moderate the expression of different kinds of aggressive behaviors (Wheeler et al., 2002; Giancola, 2003; Lovett and Sheffield, 2007; Jolliffe and Farrington, 2011) and increase prosocial behaviors (Davis et al., 1994; McMahon et al., 2006; Gini et al., 2008; Masten et al., 2011).

In our study, while the personal distress dimension was positively related with both difficulties in emotion regulation and hostility, perspective taking was negatively associated with both difficulties in emotion regulation and hostility. These results could be interpreted according to several models of emotion regulation (Rottenberg and Gross, 2003; Koole, 2009; Aldao et al., 2010) suggesting that individuals use different strategies (i.e., automatic or controlled, adaptive or maladaptive) to cope with their emotional experiences as well as to respond to environmental demands (Rottenberg and Gross, 2003; Koole, 2009; Aldao et al., 2010). It has been observed that while adaptive strategies (e.g., reappraisal and problem solving) are related with good health outcomes, dysfunctional strategies (e.g., suppression and avoidance) are associated with mental disorders and behavioral problems (Aldao et al., 2010).

The personal distress dimension assesses the tendency to experience severe discomfort in response to extreme distress in others during a tense emotional situation (Davis, 1980, 1983), and high personal distress scores has been positively associated with irritability, resentment, and suspicion (Davis, 1983). Therefore, during stressful interpersonal settings, individuals with high scores in this empathy dimension, may use hostile behaviors as a dysfunctional coping strategy to escape from that unpleasant state and/or self-regulate emotions. This is in line with several studies suggesting that subjects may engage in aggressive behaviors in order to regulate and/or improve their own affective states (Bushman, 2002). Conversely, perspective taking assess *“the tendency to spontaneously adopt the psychological point of view of others”* (Davis, 1983, pp. 113–114). Previous research reported that perspective taking was positively associated with high levels of self-esteem (Davis, 1983), as well as with prosocial behaviors (Davis et al., 1994; McMahon et al., 2006; Gini et al., 2008; Masten et al., 2011). Therefore, during stressful interpersonal situations, individuals with high scores in this empathy dimension may regulate emotion engaging in functional behaviors (e.g., prosocial behavior) rather than hostile behaviors.

It is also interesting to notice that IRI’s fantasy dimension was positively associated with difficulties in emotion regulation, but not with hostility. Previous studies reported that this dimension of empathy was positively associated with emotional vulnerability (Davis, 1983; Kawakami and Katahira, 2015) as well as with sensitivity to others, and introversion (Davis, 1983). It is known that individuals with high introversive personality, are more worried and uncertain in social situations and frequently suppress/avoid their emotions (Aldao et al., 2010; Gresham and Gullone, 2012; Vantieghem et al., 2016). Thus, according to our results (i.e., positive correlation between fantasy and DERS and no significant correlation between fantasy and BDHI), in stressful interpersonal settings, people with higher fantasy may experience difficulties in emotion regulation and use dysfunctional coping strategies, such as avoidance, rather than hostile behaviors. However, it is important to notice that our interpretation remains largely speculative because, in the present study, we did not assess coping strategies. Furthermore, the small correlation between DERS and fantasy should be considered when drawing definitive conclusions from our data.

Our mediation model indicated that more severe difficulties in emotion regulation were associated with higher hostility and that perspective taking partially counterbalanced this relationship. Conversely, personal distress does not seem to mediate the association between emotion regulation and hostility (**Figure 1**), suggesting the crucial role of perspective taking skills in our mediation model. It is also interesting to underline that in the reverse mediational model, no significant effect was observed for perspective taking. This dimension is considered a key component of empathy (Gerace et al., 2013). Mohr et al. (2007), showed that lower perspective taking scores were a crucial predictor of anger in students as well as in violent offenders. The same results were observed by Day et al. (2012), who proposed that the perspective taking ability may play a crucial role in inhibiting anger arousal and behavioral aggressions.

Our results could be interpreted in line with Decety’s (2010) model of empathy. The author reported that the development of emotion regulation, through the maturation of crucial brain areas [i.e., the anterior cingulate cortex (ACC) and prefrontal cortex (PFC)], is functionally linked to the development of executive and metacognition functions, which are closely related to the cognitive aspects of empathy (i.e., perspective taking) (Decety, 2010). Thus, as suggested by our results, it is possible that deficits in emotion regulation could lead to impairment in perspective taking, which make manifestations of hostility and aggressive behaviors more likely.

Moreover, our mediation model results may reflect several recent neuroimaging studies which detected some overlapping in the brain’s regions involved in emotion regulation, hostility, and empathy. From a neurobiological point of view, several brain regions, such as PFC, ACC, the insular cortex and the amygdala, play a crucial role in various aspects of emotion and emotion regulation (for a review see Davidson et al., 2000a; Arnsten and Rubia, 2012). It has been hypothesized (Davidson et al., 2000b) that functional and/or structural abnormalities in this brain areas (e.g., hypo-activation of PFC) or in the functional integration among them, may increase the tendency of hostile and aggressive behaviors. Coherently, recent studies have shown the involvement of these brain structures in empathy dimensions (for a review see Singer and Lamm, 2009). For example, Haas et al. (2015) reported that higher scores in perspective taking were associated with increased prefrontal cortex activity during an emotion attribution task. Furthermore, Banissy et al. (2012) showed that the perspective taking total score was positively correlated with gray matter volume of the anterior cingulate.

Although the present findings are promising, some issues which limit the generalizability of our results include: (i) a non-clinical sample; (ii) the use of self-report measures, which are known to be potentially affected by social desirability (Arnold and Feldman, 1981); (iii) the enrollment of a young adult cohort; (iv) the non-assessment of the diagnostic status/history of participants and several socioeconomic variables (e.g., income or ethnicity) which may affect the relationship among emotion regulation, hostility and empathy. Moreover, although BDHI was the dependent variable and DERS was the independent variable in our model, it is important to underline that the statistical design, we used is correlational in nature, which precludes a definitive causal interpretation of the association between these variables. Finally, we have assessed empathy using only IRI subscales. Therefore, it is possible that other general measures of empathy, such as the Empathy Quotient (Lawrence et al., 2004), might provide important insights on other mediators between difficulties in emotion regulation and hostility. Although these ideas are purely hypothetical, they might be useful in guiding future research studies with clinical and non-clinical samples and with longitudinal designs.

## Conclusion

Our results suggest that (i) high scores in difficulties in emotion regulation are strongly associated with high hostility, and (ii) this association is partially counterbalanced by high levels of perspective taking. From a clinical point of view, our results highlight the importance of those therapeutic approaches which focus on the enhancement of perspective taking in people with deficits in emotion regulation as well as with aggressive behavioral problems (i.e., Violent Offenders) (Hanson and Scott, 1995).

## Author Contributions

AC: Study design, interpretation of results, preparation of the manuscript. CI: Preparation of the manuscript, data analysis, interpretation of the results. IP: Data collection, preparation of the manuscript. CD: Data collection, preparation of the manuscript. BF: Study design, interpretation of results, preparation of the manuscript.

## Conflict of Interest Statement

The authors declare that the research was conducted in the absence of any commercial or financial relationships that could be construed as a potential conflict of interest.

## References

[B1] Albiero P. Ingoglia S. Lo Coco A. (2006). Contributo all’adattamento italiano dell’interpersonal reactivity index. *Test. Psicom. Methodol.* 13 107–125.

[B2] Aldao A. Mennin D. S. (2012). Paradoxical cardiovascular effects of implementing adaptive emotion regulation strategies in generalized anxiety disorder. *Behav. Res. Ther.* 50 122–130. 10.1016/j.brat.2011.12.004 22218164

[B3] Aldao A. Nolen-Hoeksema S. Schweizer S. (2010). Emotion-regulation strategies across psychopathology: a meta-analytic review. *Clin. Psychol. Rev.* 30 217–237. 10.1016/j.cpr.2009.11.004 20015584

[B4] American Psychiatric Association (2013). *Diagnostic and Statistical Manual of Mental Disorders - DSM-5.* Arlington, TX: American Psychiatric Publishing.

[B5] Amstadter A. (2008). Emotion regulation and anxiety disorders. *J. Anxiety Disord.* 22 211–221. 10.1016/j.janxdis.2007.02.004 17349775 PMC2736046

[B6] Arnold H. J. Feldman D. C. (1981). Social desirability response bias in self-report choice situations. *Acad. Manage. J.* 24 377–385. 10.2307/255848

[B7] Arnsten A. F. Rubia K. (2012). Neurobiological circuits regulating attention, cognitive control, motivation, and emotion: disruptions in neurodevelopmental psychiatric disorders. *J. Am. Acad. Child Adolesc. Psychiatry* 51 356–367. 10.1016/j.jaac.2012.01.008 22449642

[B8] Banissy M. J. Kanai R. Walsh V. Rees G. (2012). Inter-individual differences in empathy are reflected in human brain structure. *Neuroimage* 62 2034–2039. 10.1016/j.neuroimage.2012.05.081 22683384 PMC3778747

[B9] Barefoot J. C. Beckham J. C. Haney T. L. Siegler I. C. Lipkus I. M. (1993). Age differences in hostility among middle-aged and older adults. *Psychol. Aging* 8 3–9. 10.1037/0882-7974.8.1.3 8461112

[B10] Bendig A. W. (1962). Factor analytic scales of covert and overt hostility. *J. Consult. Psychol.* 26:200 10.1037/h0048664 13867102

[B11] Berking M. Poppe C. Luhmann M. Wupperman P. Jaggi V. Seifritz E. (2012). Is the association between various emotion-regulation skills and mental health mediated by the ability to modify emotions? Results from two cross-sectional studies. *J. Behav. Ther. Exp. Psychiatry* 43 931–937. 10.1016/j.jbtep.2011.09.009 22406495

[B12] Berking M. Whitley B. (2014). *Affect Regulation Training. A Practitioners’ Manual.* New York, NY: Springer.

[B13] Biaggio M. K. Supplee K. Curtis N. (1981). Reliability and validity of four anger scales. *J. Pers. Assess.* 45 639–648. 10.1207/s15327752jpa4506_12 7310621

[B14] Blanchard-Fields F. Stein R. Watson T. L. (2004). Age differences in emotion-regulation strategies in handling everyday problems. *J. Gerontol. B Psychol. Sci. Soc. Sci.* 59 261–269. 10.1093/geronb/59.6.P261 15576853

[B15] Bowie B. H. (2010). Understanding the gender differences in pathways to social deviancy: relational aggression and emotion regulation. *Arch. Psychiatr. Nurs.* 24 27–37. 10.1016/j.apnu.2009.04.007 20117686

[B16] Bushman B. J. (2002). Does venting anger feed or extinguish the flame? Catharsis, rumination, distraction, anger, and aggressive responding. *Pers. Soc. Psychol. Bull.* 28 724–731. 10.1177/0146167202289002

[B17] Bushman B. J. Cooper H. M. Lemke K. M. (1991). Meta-analysis of factor analyses: an illustration using the Buss-Durkee Hostility Inventory. *Pers. Soc. Psychol. Bull.* 17 344–349. 10.1177/0146167291173015

[B18] Buss A. H. Durkee A. (1957). An inventory for assessing different kinds of hostility. *J. Consult. Psychol.* 21 343–349. 10.1037/h0046900 13463189

[B19] Carrère S. Bowie B. H. (2012). Like parent, like child: parent and child emotion dysregulation. *Arch. Psychiatr. Nurs.* 26 e23–e30. 10.1016/j.apnu.2011.12.008 22633588

[B20] Castrogiovanni P. Maremmani I. Di Muro A. Nistico‘G. Akiskal H. S. (1993). “Aggressive behaviour and hostility in depression: clinical aspects,” in *Recurrent Mood Disorders* eds Placidi G. F. Dell’osso L. (Berlin: Springer-Verlag) 51–65.

[B21] Christie K. A. Burke J. D. Jr. Regier D. A. Rae D. S. Boyd J. H. Locke B. Z. (1988). Epidemiologic evidence for early onset of mental disorders and higher risk of drug abuse in young adults. *Am. J. Psychiatry* 145 971–975. 10.1176/ajp.145.8.971 3394882

[B22] Contardi A. Farina B. Fabbricatore M. Tamburello S. Scapellato P. Penzo I. (2013). [Difficulties in emotion regulation and personal distress in young adults with social anxiety]. *Riv. Psichiatr.* 48 155–161. 10.1708/1272.14040 23748726

[B23] Davidson K. Hall P. (1995). What does potential for hostility measure? Gender differences in the expression of hostility. *J. Behav. Med.* 18 233–247. 10.1007/BF01857871 7674290

[B24] Davidson R. J. Jackson D. C. Kalin N. H. (2000a). Emotion, plasticity, context, and regulation: perspectives from affective neuroscience. *Psychol. Bull.* 126 890–909. 10.1037/0033-2909.126.6.890 11107881

[B25] Davidson R. J. Putnam K. M. Larson C. L. (2000b). Dysfunction in the neural circuitry of emotion regulation–a possible prelude to violence. *Science* 289 591–594. 10.1126/science.289.5479.591 10915615

[B26] Davis M. H. (1980). A multidimensional approach to individual differences in empathy. *JSAS Catalog Sel. Doc. Psychol.* 10:85.

[B27] Davis M. H. (1983). Measuring individual differences in empathy: evidence for a multidimensional approach. *J. Pers. Soc. Psychol.* 44 113–126. 10.1037/0022-3514.44.1.113

[B28] Davis M. H. (1994). *Empathy: A Social Psychological Approach.* Madison, WI: Brown & Benchmark.

[B29] Davis M. H. Luce C. Kraus S. J. (1994). The heritability of characteristics associated with dispositional empathy. *J. Pers.* 62 369–391. 10.1111/j.1467-6494.1994.tb00302.x 7965564

[B30] Day A. Mohr P. Howells K. Gerace A. Lim L. (2012). The role of empathy in anger arousal in violent offenders and university students. *Int. J. Offender Ther. Comp. Criminol.* 56 599–613. 10.1177/0306624X11431061 22158909

[B31] De Corte K. Buysse A. Verhofstadt L. L. Roeyers H. Ponnet K. Davis M. H. (2007). Measuring empathic tendencies: reliability and validity of the Dutch version of the interpersonal reactivity index. *Psychol. Belg.* 47 235–260. 10.5334/pb-47-4-235

[B32] de Wied M. Gispen-De Wied C. Van Boxtel A. (2010). Empathy dysfunction in children and adolescents with disruptive behavior disorders. *Eur. J. Pharmacol.* 626 97–103. 10.1016/j.ejphar.2009.10.016 19836371

[B33] Decety J. (2010). The neurodevelopment of empathy in humans. *Dev. Neurosci.* 32 257–267. 10.1159/000317771 20805682 PMC3021497

[B34] Eisenberg N. Fabes R. A. Murphy B. Karbon M. Maszk P. Smith M. (1994). The relations of emotionality and regulation to dispositional and situational empathy-related responding. *J. Pers. Soc. Psychol.* 66 776–797. 10.1037/0022-3514.66.4.776 8189352

[B35] Fernández A. M. Dufey M. Kramp U. (2011). Testing the psychometric properties of the interpersonal reactivity index (IRI) in Chile. *Eur. J. Psychol. Assess.* 27 179–185. 10.1027/1015-5759/a000065

[B36] Fossati A. Barratt E. S. Carretta I. Leonardi B. Grazioli F. Maffei C. (2004). Predicting borderline and antisocial personality disorder features in nonclinical subjects using measures of impulsivity and aggressiveness. *Psychiatry Res.* 125 161–170. 10.1016/j.psychres.2003.12.001 15006439

[B37] Garcia-Leon A. Reyes G. A. Vila J. Perez N. Robles H. Ramos M. M. (2002). The aggression questionnaire: a validation study in student samples. *Span. J. Psychol.* 5 45–53. 10.1017/S1138741600005825 12025365

[B38] Gerace A. Day A. Casey S. Mohr P. (2013). An exploratory investigation of the process of perspective taking in interpersonal situations. *J. Relatsh. Res.* 4 1–12.

[B39] Giancola P. R. (2003). The moderating effects of dispositional empathy on alcohol-related aggression in men and women. *J. Abnorm. Psychol.* 112 275–281. 10.1037/0021-843X.112.2.275 12784837

[B40] Gillanders S. Wild M. Deighan C. Gillanders D. (2008). Emotion regulation, affect, psychosocial functioning, and well-being in hemodialysis patients. *Am. J. Kidney Dis.* 51 651–662. 10.1053/j.ajkd.2007.12.023 18371541

[B41] Gini G. Albiero P. Benelli B. Altoe G. (2007). Does empathy predict adolescents’ bullying and defending behavior? *Aggress. Behav.* 33 467–476. 10.1002/ab.20204 17683107

[B42] Gini G. Albiero P. Benelli B. Altoe G. (2008). Determinants of adolescents’ active defending and passive bystanding behavior in bullying. *J. Adolesc.* 31 93–105. 10.1016/j.adolescence.2007.05.002 17574660

[B43] Giromini L. Velotti P. De Campora G. Bonalume L. Cesare Zavattini G. (2012). Cultural adaptation of the difficulties in emotion regulation scale: reliability and validity of an Italian version. *J. Clin. Psychol.* 68 989–1007. 10.1002/jclp.21876 22653763

[B44] Gratz K. L. Roemer L. (2004). Multidimensional assessment of emotion regulation and dysregulation: development, factor structure, and initial validation of the difficulties in emotion regulation scale. *J. Psychopathol. Behav. Assess.* 26 41–54. 10.1023/B:JOBA.0000007455.08539.94

[B45] Gresham D. Gullone E. (2012). Emotion regulation strategy use in children and adolescents: the explanatory roles of personality and attachment. *Pers. Individ. Dif.* 52 616–621. 10.1016/j.paid.2011.12.016

[B46] Haas B. W. Anderson I. W. Filkowski M. M. (2015). Interpersonal reactivity and the attribution of emotional reactions. *Emotion* 15 390–398. 10.1037/emo0000053 25706827

[B47] Hanson R. K. Scott H. (1995). Assessing perspective-taking among sexual offenders, nonsexual criminals, and nonoffenders. *Sex Abuse* 7 259–277.

[B48] Hayes A. F. (2013). *Introduction to Mediation, Moderation, and Conditional Process Analysis: A Regression Based Approach.* New York, NY: The Guilford Press.

[B49] Ingoglia S. Lo Coco A. Albiero P. (2016). Development of a brief form of the interpersonal reactivity index (B-IRI). *J. Pers. Assess.* 1–11. 10.1080/00223891.2016.1149858 [Epub ahead of print]. 27050826

[B50] Jimenez S. S. Niles B. L. Park C. L. (2010). A mindfulness model of affect regulation and depressive symptoms: positive emotions, mood regulation expectancies, and self-acceptance as regulatory mechanisms. *Pers. Individ. Dif.* 49 645–650. 10.1016/j.paid.2010.05.041

[B51] Jolliffe D. Farrington D. P. (2004). Empathy and offending: a systematic review and meta-analysis. *Aggress. Violent Behav.* 9 441–476. 10.1016/j.avb.2003.03.001

[B52] Jolliffe D. Farrington D. P. (2011). Is low empathy related to bullying after controlling for individual and social background variables? *J. Adolesc.* 34 59–71. 10.1016/j.adolescence.2010.02.001 20202677

[B53] Jones P. B. (2013). Adult mental health disorders and their age at onset. *Br. J. Psychiatry Suppl.* 54 s5–s10. 10.1192/bjp.bp.112.119164 23288502

[B54] Kawakami A. Katahira K. (2015). Influence of trait empathy on the emotion evoked by sad music and on the preference for it. *Front. Psychol.* 6:1541 10.3389/fpsyg.2015.01541 PMC4621277 26578992

[B55] Koole S. L. (2009). The psychology of emotion regulation: an integrative review. *Cogn. Emot.* 23 4–41. 10.1080/02699930802619031

[B56] Lawrence E. J. Shaw P. Baker D. Baron-Cohen S. David A. S. (2004). Measuring empathy: reliability and validity of the empathy quotient. *Psychol. Med.* 34 911–919. 10.1017/S0033291703001624 15500311

[B57] Lovett B. J. Sheffield R. A. (2007). Affective empathy deficits in aggressive children and adolescents: a critical review. *Clin. Psychol. Rev.* 27 1–13. 10.1016/j.cpr.2006.03.003 16697094

[B58] Marroquín B. (2011). Interpersonal emotion regulation as a mechanism of social support in depression. *Clin. Psychol. Rev.* 31 1276–1290. 10.1016/j.cpr.2011.09.005 21983267

[B59] Masten C. L. Morelli S. A. Eisenberger N. I. (2011). An fMRI investigation of empathy for ’social pain’ and subsequent prosocial behavior. *Neuroimage* 55 381–388. 10.1016/j.neuroimage.2010.11.060 21122817

[B60] Matthews K. A. Jamison J. W. Cottington E. M. (1985). “Assessment of Type A, anger and hostility: a review of scales through 1982” in *Measuring Psychosocial Variables in Epidemiologic Studies of Cardiovascular Disease* eds Ostfeld A. M. Eaker E. D. (Bethesda, MD: National Institutes of Health).

[B61] McLaughlin K. A. Hatzenbuehler M. L. Mennin D. S. Nolen-Hoeksema S. (2011). Emotion dysregulation and adolescent psychopathology: a prospective study. *Behav. Res. Ther.* 49 544–554. 10.1016/j.brat.2011.06.003 21718967 PMC3153591

[B62] McMahon S. D. Wernsman J. Parnes A. L. (2006). Understanding prosocial behavior: the impact of empathy and gender among African American adolescents. *J. Adolesc. Health* 39 135–137. 10.1016/j.jadohealth.2005.10.008 16781977

[B63] Mennin D. S. Holaway R. M. Fresco D. M. Moore M. T. Heimberg R. G. (2007). Delineating components of emotion and its dysregulation in anxiety and mood psychopathology. *Behav. Ther.* 38 284–302. 10.1016/j.beth.2006.09.001 17697853

[B64] Mitrofan N. Ciuluvică C. (2012). Anger and hostility as indicators of emotion regulation and of the life satisfaction at the beginning and the ending period of the adolescence. *Procedia Soc. Behav. Sci.* 33 65–69. 10.1016/j.sbspro.2012.01.084

[B65] Mohr P. Howells K. Gerace A. Day A. Wharton M. (2007). The role of perspective taking in anger arousal. *Pers. Individ. Dif.* 43 507–517. 10.1016/j.paid.2006.12.019

[B66] Musante L. Macdougall J. M. Dembroski T. M. Costa P. T. Jr. (1989). Potential for hostility and dimensions of anger. *Health Psychol.* 8 343–354. 10.1037/0278-6133.8.3.343 2767023

[B67] Nolen-Hoeksema S. (2012). Emotion regulation and psychopathology: the role of gender. *Annu. Rev. Clin. Psychol.* 8 161–187. 10.1146/annurev-clinpsy-032511-143109 22035243

[B68] Nolen-Hoeksema S. Wisco B. E. Lyubomirsky S. (2008). Rethinking rumination. *Perspect. Psychol. Sci.* 3 400–424. 10.1111/j.1745-6924.2008.00088 26158958

[B69] Okun M. A. Shepard S. A. Eisenberg N. (2000). The relations of emotionality and regulation to dispositional empathy-related responding among volunteers-in-training. *Pers. Individ. Dif.* 28 367–382. 10.1016/S0191-8869(99)00107-5

[B70] Pedersen R. (2009). Empirical research on empathy in medicine-A critical review. *Patient Educ. Couns.* 76 307–322. 10.1016/j.pec.2009.06.012 19631488

[B71] Pompili M. Innamorati M. Lamis D. A. Lester D. Di Fiore E. Giordano G. (2015). The interplay between suicide risk, cognitive vulnerability, subjective happiness and depression among students. *Curr. Psychol.* 1–16. 10.1007/s12144-015-9313-2

[B72] Preacher K. J. Hayes A. F. (2008). Asymptotic and resampling strategies for assessing and comparing indirect effects in multiple mediator models. *Behav. Res. Methods* 40 879–891. 10.3758/BRM.40.3.879 18697684

[B73] Roberton T. Daffern M. Bucks R. S. (2012). Emotion regulation and aggression. *Aggress. Violent Behav.* 17 72–82. 10.1016/j.avb.2011.09.006

[B74] Roll J. Koglin U. Petermann F. (2012). Emotion regulation and childhood aggression: longitudinal associations. *Child Psychiatry Hum. Dev.* 43 909–923. 10.1007/s10578-012-0303-4 22528031

[B75] Rottenberg J. Gross J. J. (2003). When emotion goes wrong: realizing the promise of affective science. *Clin. Psychol. Sci. Pract.* 10 227–232. 10.1093/clipsy.bpg012

[B76] Salthouse T. A. (2011). All data collection and analysis methods have limitations: reply to Rabbitt (2011) and Raz and Lindenberger (2011). *Psychol. Bull.* 137 796–799. 10.1037/a0024843 21859180 PMC3160711

[B77] Sarason I. G. (1961). Intercorrelations among measures of hostility. *J. Clin. Psychol.* 17 192–195. 10.1002/1097-4679 13746472

[B78] Sighinolfi C. Norcini Pala A. Chiri L. R. Marchetti I. Sica C. (2010). Difficulties in emotion regulation scale (DERS): traduzione e adattamento italiano. *Psicote. Cogn. Comp.* 16 141–170.

[B79] Singer T. Lamm C. (2009). The social neuroscience of empathy. *Ann. N. Y. Acad. Sci.* 1156 81–96. 10.1111/j.1749-6632.2009.04418 19338504

[B80] Spreng R. N. Mckinnon M. C. Mar R. A. Levine B. (2009). The Toronto Empathy Questionnaire: scale development and initial validation of a factor-analytic solution to multiple empathy measures. *J. Pers. Assess.* 91 62–71. 10.1080/00223890802484381 19085285 PMC2775495

[B81] Steinberg E. A. Drabick D. A. (2015). A developmental psychopathology perspective on ADHD and comorbid conditions: the role of emotion regulation. *Child Psychiatry Hum. Dev.* 46 951–966. 10.1007/s10578-015-0534-2 25662998

[B82] Svaldi J. Griepenstroh J. Tuschen-Caffier B. Ehring T. (2012). Emotion regulation deficits in eating disorders: a marker of eating pathology or general psychopathology? *Psychiatry Res.* 197 103–111. 10.1016/j.psychres.2011.11.009 22401969

[B83] Thompson R. A. (1994). Emotion regulation: a theme in search of definition. *Monogr. Soc. Res. Child Dev.* 59 25–52. 10.1111/j.1540-5834.1994.tb01276.x 7984164

[B84] Vantieghem I. Marcoen N. Mairesse O. Vandekerckhove M. (2016). Emotion regulation mediates the relationship between personality and sleep quality. *Psychol. Health* 31 1–16. 10.1080/08870446.2016.1171866 27021392

[B85] Wheeler J. G. George W. H. Dahl B. J. (2002). Sexually aggressive college males: empathy as a moderator in the “Confluence Model” of sexual aggression. *Pers. Individ. Dif.* 33 759–775. 10.1016/S0191-8869(01)00190-8

